# A Genetic Screen Reveals Novel Targets to Render *Pseudomonas aeruginosa* Sensitive to Lysozyme and Cell Wall-Targeting Antibiotics

**DOI:** 10.3389/fcimb.2017.00059

**Published:** 2017-03-01

**Authors:** Kang-Mu Lee, Keehoon Lee, Junhyeok Go, In Ho Park, Jeon-Soo Shin, Jae Young Choi, Hyun Jik Kim, Sang Sun Yoon

**Affiliations:** ^1^Department of Microbiology and Immunology, Yonsei University College of MedicineSeoul, South Korea; ^2^Brain Korea 21 PLUS Project for Medical Sciences, Yonsei University College of MedicineSeoul, South Korea; ^3^Department of Microbiology and Immunology, Severance Biomedical Science Institute, Yonsei University College of MedicineSeoul, South Korea; ^4^Department of Microbiology and Immunology, Institute for Immunology and Immunological Diseases, Yonsei University College of MedicineSeoul, South Korea; ^5^Department of Otorhinolaryngology, Yonsei University College of MedicineSeoul, South Korea; ^6^Department of Otorhinolaryngology, Seoul National University College of MedicineSeoul, South Korea

**Keywords:** *Pseudomonas aeruginosa*, lysozyme, treatment regimen, airway infection, multi-drug resistance

## Abstract

*Pseudomonas aeruginosa* is capable of establishing airway infections. Human airway mucus contains a large amount of lysozyme, which hydrolyzes bacterial cell walls. *P. aeruginosa*, however, is known to be resistant to lysozyme. Here, we performed a genetic screen using a mutant library of PAO1, a prototype *P. aeruginosa* strain, and identified two mutants (Δ*bamB* and Δ*fabY*) that exhibited decrease in survival after lysozyme treatment. The *bamB* and *fabY* genes encode an outer membrane assembly protein and a fatty acid synthesis enzyme, respectively. These two mutants displayed retarded growth in the airway mucus secretion (AMS). In addition, these mutants exhibited reduced virulence and compromised survival fitness in two different *in vivo* infection models. The mutants also showed susceptibility to several antibiotics. Especially, Δ*bamB* mutant was very sensitive to vancomycin, ampicillin, and ceftazidime that target cell wall synthesis. The Δ*fabY* displayed compromised membrane integrity. In conclusion, this study uncovered a common aspect of two different *P. aeruginosa* mutants with pleiotropic phenotypes, and suggests that BamB and FabY could be novel potential drug targets for the treatment of *P. aeruginosa* infection.

## Introduction

Antibiotic-resistant *P. aeruginosa* strains are emerging at a significantly faster rate than the introduction of new anti-Pseudomonal agents (Pendleton et al., 2013). Furthermore, prolonged use of antibiotics has been proven to be a critical risk factor in the selection of multi-drug resistant variants, especially in the case of *P. aeruginosa* infection (Merlo et al., 2007). It is, therefore, crucial to devise a revolutionary strategy to combat *P. aeruginosa* infection. One such way would be to take advantage of the host innate defense molecules. Since the early discovery of the inherent lysozyme resistance of *P. aeruginosa* (Warren et al., 1955), no significant efforts have been made to increase the susceptibility of *P. aeruginosa* to lysozyme.

Lysozyme is an important antibacterial protein that is abundantly present in the human airway (Duszyk, 2001; Dubin et al., 2004; Dajani et al., 2005). Its enzymatic activity is to cleave β-1,4-glycosidic linkages on the N-acetylglucosamine (NAG) and N-acetylmuramic acid (NAM) polysaccharide chains within the bacterial peptidoglycan layer (Vanderwinkel et al., 1995). Lysozyme found in the human airway is more active than egg-white lysozyme due to distinct structural differences between the two molecules (Marx et al., 1986). Although lysozyme has been proposed to play a crucial role in host defense, persistent *P. aeruginosa* airway infection is a major cause of morbidity and mortality in cystic fibrosis (CF) patients. Moreover, higher lysozyme activity has been detected in the serum and saliva of CF patients compared with normal individuals (Hughes et al., 1982). Likewise, lysozyme activity was also higher in bronchoalveolar lavage fluid (BALF) collected from CF patients (Sagel et al., 2009). Together, these results suggest that human lysozyme is ineffective in killing *P. aeruginosa* during infection. The lack of lysozyme effectiveness against *P. aeruginosa* has also been attributed to its active production of elastase, a major metalloprotease that can cleave human lysozyme (Jacquot et al., 1985).

Besides the infection in CF patients, *P. aeruginosa* is one of the major pathogens that cause ventilator-associated pneumonia (VAP). Especially, the pneumonia and sepsis by *P. aeruginosa* are the serious threats to the cardiac or thoracic surgical patients and trauma patients in the Intensive Care Unit (ICU; Berra et al., 2010). *P. aeruginosa* can also cause acute lung injury by injecting the ExoU toxin into the cytosol of eukaryotic cells using Type III Secretion System (TTSS; Pankhaniya et al., 2004). The acute *P. aeruginosa* infection could disrupt the alveolar epithelial barrier to promote necrosis of lung epithelial cells and sepsis, leading rapid increase in mortality (Sawa, 2014).

In this study, we aimed to identify novel interventional approaches that increase the sensitivity of *P. aeruginosa* to lysozyme. To this end, we isolated *P. aeruginosa* mutants that lost their viability when treated with lysozyme and sought to describe the genetic and phenotypic bases of their lysozyme sensitivity. Such defective mutants were found to be incapable of propagating in airway mucus secretions (AMSs) or establishing infection in the mouse airway, two locations where lysozyme is present in high concentrations. Results provided here will stimulate future works to propose novel approaches for inhibiting *P. aeruginosa* growth under lysozyme-rich environments.

## Materials and methods

### Experimental ethics

Experiments using human subjects and experimental animals were performed in strict accordance with guidelines provided by Yonsei University. Protocols were reviewed and approved by Institutional Review Board of Yonsei University College of Medicine. Permit numbers for primary culture of human tissues and mouse infection experiment were 2014-1842-001 and 2013-0369-5, respectively.

### Bacterial strains and growth conditions

**Figure 1:**
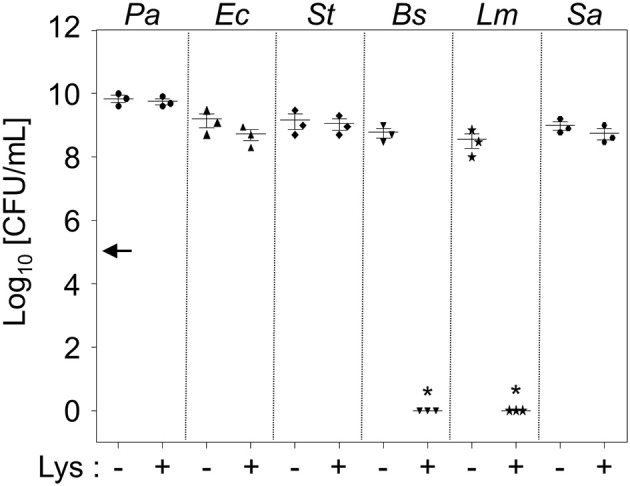
**Intrinsic resistance of *P. aeruginosa* to lysozyme**. Six different bacterial species were treated with 1 mg/mL lysozyme in 0.5 × LB for 16 h. Bacterial cells were enumerated by plating serially-diluted cells on LB agar plates. Each culture contained an initial inoculum of 1 × 10^5^ cells. Bacterial cultures were repeated in triplicate, and the mean ± SD values are displayed. ^*^P < 0.05 vs. CFU of untreated cultures.

A prototype strain of *P. aeruginosa* called PAO1 was used in this study (Yoon et al., 2006). The bacterial strains of the other species, as listed in Figure 1, were obtained from our laboratory stock and were previously reported (Gi et al., 2015). Bacterial cultures were grown in Luria-Bertani medium (LB; 10 g tryptone, 5 g yeast extract, and 10 g NaCl/l), unless otherwise stated. Bacterial cells were cultured at 37°C for 16 h and shaken at 200 rpm. Bacterial growth in AMS and survival following the treatments with diverse antibiotics were assessed by quantifying colony forming units (CFUs).

### Mutant library screen for lysozyme-sensitive mutants and preparation of airway mucus secretion (AMS)

A transposon (Tn) insertion mutant library of PAO1 was constructed following the procedures described elsewhere (Lee et al., 2016). In brief, PAO1 was conjugated with *Escherichia coli* SM10/λpir harboring the pBTK30 plasmid (Table 1). Gentamicin (Gm)-resistant transconjugants were grown on LB agar plates containing 50 μg/ml Gm and 50 μg/ml Irgasan (Sigma). Irgasan was added to eliminate *E. coli* donor cells. To isolate lysozyme-sensitive mutants, each individual mutant was grown in 0.5 × LB media (50% LB + 50% distilled water) containing 1 mg/mL lysozyme (Sigma) in 96 well plates. Bacterial growth was monitored by measuring the OD_600_. The Tn insertion site of each defective mutant was determined by arbitrary PCR, followed by DNA sequencing (Lee et al., 2016). Preparation of AMS samples from Normal Healthy Trachea Epithelial (NHTE) cells and bacterial growth in AMS were conducted as previously described (Gi et al., 2015).

**Table 1:** **Bacterial strains and plasmids used in this study**.

**Strains or plasmids**	**Relevant characteristics**	**References or source**
***P. aeruginosa*** **strains**		
PAO1	Standard lab strain	Yoon et al., 2006
Δ*PA0420*	PA01, *PA0420* gene deleted	This study
Δ*PA3800*	PAO1, *PA3800* gene deleted	This study
Δ*PA5174*	PAO1, *PA5174* gene deleted	This study
Δ*PA3800*/pJN105	Δ*PA3800* harboring pJN105	This study
Δ*PA5174*/pJN105	Δ*PA5174* harboring pJN105	This study
Δ*PA3800*/pJN105::*PA3800*	Δ*PA3800* harboring pJN105::*PA3800*	This study
Δ*PA5174*/pJN105::*PA5174*	Δ*PA5174* harboring pJN105::*PA5174*	This study
***E. coli*** **strains**		
SM10/λpir	Km^r^*thi-1 thr leu tonA lacY supE recA*::RP4–2-Tc::Mu *pir^+^*, for conjugal transfer	Simon et al., 1983
**OTHER BACTERIAL SPECIES**		
*Staphylococcus aureus*	ATCC 29213	Lab collection
*Bacillus subtilis*	ATCC 6633	Lab collection
*Escherichia coli*	ATCC 25922	Lab collection
*Salmonella typhimurium*	LT2 strain	Lab collection
*Listeria monocytogenes*	ATCC 19111	Lab collection
**PLASMIDS**		
pBTK30	Transposon vector for construction of a random mutant library, Gm^r^	Kim et al., 2012
pCVD442	*sacB* suicide vector derived from plasmid pUM24	Lee et al., 2012
pJN105	*araC*-PBAD cassette cloned in pBBR1MCS-5, Gm^r^	Newman and Fuqua, 1999
pJN105::*PA3800*	pJN105 with wild-type copy of *PA3800* gene under arabinose-inducible promoter	This study
pJN105::*PA5174*	pJN105 with wild-type copy of *PA5174* gene under arabinose-inducible promoter	This study

### Construction of the *PA0420, PA3800*, and *PA5174* clean deletion mutants

The *bioA, bamB*, and *fabY* deletion mutants were created by allelic replacement as previously described (Lee et al., 2012). Briefly, flanking sequences (~600 bp) at both ends of each gene were PCR amplified with primers listed in Table 2. Two inner primers (upstream reverse primer and downstream forward primer) are complementary to each other. In this strategy, the 3′-end of the upstream sequence and the 5′-end of the downstream sequence are annealed during PCR amplification without further treatment. The deletion of the *PA0420, PA3800*, and *PA5174* genes was confirmed by PCR and DNA sequencing.

**Table 2:** **Primers used in this study**.

**Gene name**	**Direction**	**Primer sequence (5′−3′)^a^**	**Restriction enzymes**
**MUTANT CONSTRUCTION**			
*PA0420* left	Forward	ACCTTGAGCTCGCCGAGAGATCGTCCAGGGT	*SacI*
*PA0420* left	Reverse	TCCGGGTGGAAGTCGCTGACAAGGCCCATGGGCTGTCTCC	
*PA0420* right	Forward	GGAGACAGCCCATGGGCCTTGTCAGCGACTTCCACCCGGA	
*PA0420* right	Reverse	TAGAGGAGCTCAGGGCCAGAGGCGGATGGAT	*SacI*
*PA3800* left	Forward	ACCTTGCATGCTGGTGGCAGCGCAACGGCAA	*SphI*
*PA3800* left	Reverse	ACGAGCTTGCCACCGTTGCCCACCATCTCAGGCCTCTCCC	
*PA3800* right	Forward	GGGAGAGGCCTGAGATGGTGGGCAACGGTGGCAAGCTCGT	
*PA3800* right	Reverse	TAGAGGCATGCGATCCCCAGGGCTTCCTGCA	*SphI*
*PA5174* left	Forward	ACCTTGAGCTCGTCCAGGCCGCCATCGAGTT	*SacI*
*PA5174* left	Reverse	GGCGCTCAGTCGAGCATGTCGCCTGCTGCGTTGTAGCCAC	
*PA5174* right	Forward	GTGGCTACAACGCAGCAGGCGACATGCTCGACTGAGCGCC	
*PA5174* right	Reverse	TAGAGGAGCTCCCGGTAGCGAGGAGTTCACC	*SacI*
**COMPLEMENTATION**			
*PA3800* complementation (cloning to pJN105)	Forward	AATTCGAATTCAAGGAGATATACATATGGTGCAATGGAAACACGC	*EcoRI*
	Reverse	ATATCTCTAGACTAGCGGATGGTGTAGGCGA	*XbaI*
*PA5174* complementation (cloning to pJN105)	Forward	AATTCGAATTCAAGGAGATATACATATGTCTCGACTACCGGTCATT	*EcoRI*
	Reverse	ATATCTCTAGATCAGTCGAGCATGTCGCTGA	*XbaI*

a *Restriction enzyme recognition sequences are underlined*.

### Genetic complementation of the Δ*PA3800* and Δ*PA5174* mutants

To complement the Δ*PA3800* and Δ*PA5174*, DNA fragments containing the entire *PA3800* and *PA5174* genes were amplified from the PAO1 genome and ligated into *EcoRI*/*XbaI*-treated pJN105. The resultant plasmid and the control empty plasmid (i.e., pJN105) were transferred into the Δ*PA3800* and Δ*PA5174* mutant by electroporation, respectively. Expression of *PA3800* or *PA5174* gene is induced by 0.01% L-arabinose (Sigma).

### Bacterial growth in airway mucus secretion (AMS)

Bacterial cultures grown in LB for 8 h were diluted in PBS to get bacterial suspensions with the 10^5^ CFU/mL. Ten microliters of each diluent was inoculated into 100 μL AMS to achieve the initial inoculum size of ~10^3^ CFU in AMS. Bacterial cells were then grown for 16 h in a humidified 37°C incubator. Bacterial growth was assessed by measuring the growth index. The values for [CFU after 16 h in AMS / CFU after 0 h in AMS] were calculated and plotted as the growth index.

### Mouse infection and *Caenorhabditis elegans* survival test

Animal experiments were approved by the Committee on the Ethics of Animal Experiments of Yonsei University College of Medicine (IACUC permit number: 2013-0369-5). Animal experiments were conducted following national guidelines provided by the Korean government (Ministry for Food, Agriculture, Forestry and Fisheries) and in strict accordance with the institutional guidelines for animal care and use of laboratory animals. To test the virulence of the Δ*bamB* and Δ*fabY* mutants *in vivo*, 8-week-old C57BL/6N inbred female mice (Orient, Korea) were intranasally infected with 5 × 10^6^ cells of each strain (*n* = 4). The lungs of the infected mice were removed at 16 h post-infection. The harvested lungs were homogenized, and the number of bacteria in each organ was measured by quantifying CFUs. *Caenorhabditis elegans* survival tests were performed as described previously (Go et al., 2014).

### Electron microscope

Lysozyme-treated bacterial cells were visualized using a scanning electron microscope (SEM), as described previously (Yoon et al., 2011). Briefly, fixed bacterial suspensions were stained with 1% OsO4 (Sigma) and then coated with gold via an ion sputter (IB-3 Eiko, Japan). SEM (FE SEM S-800, Hitachi, Japan) was used at an acceleration voltage of 20 kV. All images were processed using ESCAN 4000 software (Bummi Universe Co., Ltd., Seoul, Korea).

### Membrane integrity assays

Bacterial resistance against hypo-osmotic stress was measured by monitoring the decrease of OD_600_ values. Each bacterial strain, grown to mid-exponential phase was harvested and washed with 1 × PBS. The bacterial cells were resuspended in sterilized deionized water. The OD_600_ of each bacterial suspension was measured every 10 min for 60 min. The data were normalized with their initial OD_600_-values. The PAO1, Δ*bamB*, and Δ*fabY* mutants were also observed under confocal laser scanning microscope (CLSM) with LIVE/DEAD® *Bac*Light™ Bacterial Viability Kit to detect the difference in membrane integrity among these bacterial strains.

### Statistical analysis

The experiments were repeated at least in triplicate, and data are expressed as mean ± standard deviation (*SD*). Data were analyzed using unpaired Student's *t*-test, unless otherwise stated and *P* < 0.05 were considered to be statistically significant. Log-rank test was used to provide statistical significance in the *C. elegans* lifespan experiments.

## Results

### *P. aeruginosa* is highly resistant to lysozyme

Prior literature demonstrated that *P. aeruginosa* can become lysozyme-sensitive only when co-treated with EDTA at pH 8.0 (Voss, 1964) or pretreated with acetone or heat (Warren et al., 1955). As expected, PAO1 grew unencumbered when grown in LB media supplemented with 1 mg/mL lysozyme (Figure 1). Likewise, two other Gram-negative species, *Escherichia coli* (*Ec*) and *Salmonella enterica serovar Typhimurium (St)*, also grew normally in these conditions (Figure 1). These results further support our notion that Gram-negative bacterial cells are intrinsically resistant to lysozyme because their outer membrane prevents lysozyme from accessing the peptidoglycan in the periplasm. On the other hand, two Gram-positive species, *Bacillus subtilis* (*Bs*) and *Listeria monocytogenes* (*Lm*), lost their viability in response to the same treatment (Figure 1). Consistent with previous reports (Bera et al., 2005, 2006), *Staphylococcus aureus* (*Sa*) was completely resistant to the lysozyme treatment (Figure 1). PAO1 growth was not affected, even in the presence of 8 mg/mL lysozyme (data not shown), demonstrating an exceptionally high resistance to lysozyme.

### Dentification of lysozyme-sensitive PAO1 mutants

**Figure 2:**
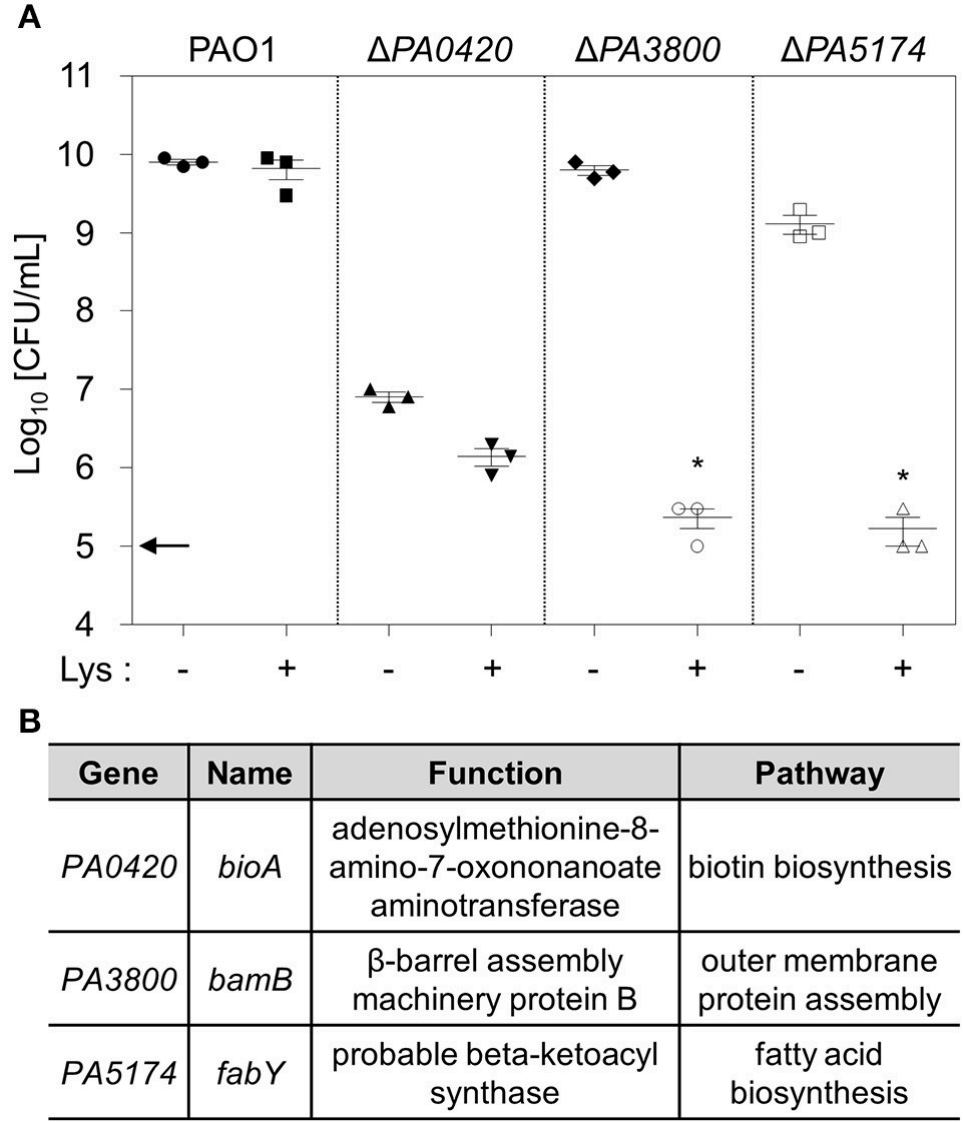
**Identification of lysozyme-sensitive PAO1 mutants. (A)** Three PAO1 mutant strains were grown in the absence or presence of 1 mg/mL lysozyme in 0.5 × LB. After 16 h growth, aliquots from each culture were diluted and plated for CFU counting. Experimental conditions were identical to those described in the legend of Figure 1. Bacterial cultures were repeated in triplicate, and the mean ± SD values are displayed. ^*^P < 0.05 vs. CFU of untreated cultures. **(B)** Genetic information for the genes disrupted in each mutant and the functions of the proteins they encoded.

In order to devise a better strategy for *P. aeruginosa* infection control, it would be desirable to identify lysozyme-sensitive mutants. We therefore constructed a Tn insertion mutant library and screened it for mutants that had lost their resistance to lysozyme treatment. Since lysozyme is more active in environments with reduced ionic strength (Sorrentino et al., 1982; Verhamme et al., 1988), bacterial growth was tested in two-fold diluted LB media (termed 0.5 × LB). Approximately 5,500 mutants were screened, three of which were determined to lack lysozyme resistance. In each mutant, the transposon insertion occurred in *PA0420, PA3800*, or *PA5174* gene. To further verify the effects of these gene disruptions, we generated an in-frame deletion of each gene and examined cell growth in the presence of lysozyme. In 0.5 × LB, Δ*PA3800* and Δ*PA5174* mutants exhibited significant growth inhibition in the presence of 1.0 mg/mL lysozyme (Figure 2A). After 16 h of growth at 37°C with lysozyme, the CFUs of these two mutants remained similar to those at the time of inoculum (indicated by an arrow on the y-axis), indicating that bacterial growth was prevented by lysozyme at a physiological concentration. *PA3800* gene encode β-barrel assembly machinery protein B (BamB) involved in outer membrane protein assembly (Jansen et al., 2012). *PA5174* gene encodes probable beta-ketoacyl synthase, which plays a role in fatty acid biosynthesis (Yuan et al., 2012; Figure 2B). The deletion of *PA3800* gene did not result in any growth inhibition, whereas the Δ*fabY* mutant exhibited a slightly affected growth, yielding ~10-fold less viable cell counts after 16 h growth in 0.5 × LB media (Figure 2A). When these mutants were grown with lysozyme, bacterial cells with crumbled rod-shaped morphology were observed (Figure S1), further demonstrating that lysozyme, an enzyme that degrades peptidoglycan polymers, damages the bacterial cell wall structure.

*PA0420, bioA* gene, produces adenosylmethionine-8-amino-7-oxononanoate aminotransferase, which is responsible for biotin biosynthesis (Beaume et al., 2015). Of note, the Δ*bioA* mutant exhibited inactive growth, even in the absence of lysozyme treatment, indicating that an interruption in biotin synthesis leads to defective growth in *P. aeruginosa*. When the mutant was grown in media supplemented with extraneous biotin, its growth and lysozyme susceptibility were completely restored (Figure S2). This result suggests that lysozyme sensitivity of the Δ*bioA* mutant is likely associated with its faulty growth; therefore, we did not pursue any further investigation on this particular mutant.

**Figure 3:**
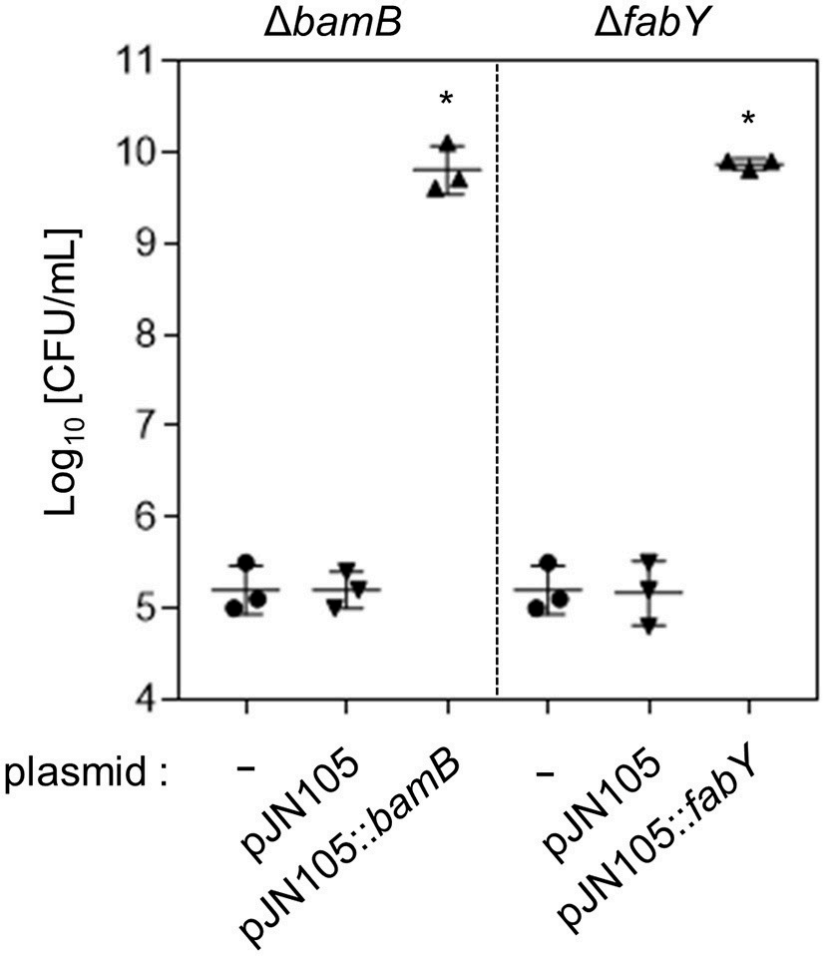
**Restored lysozyme resistance of genetically complemented mutants**. The Δ*bamB* and Δ*fabY* mutants were transformed with pJN105::*bamB* or pJN105::*fabY* plasmid, respectively. As a control, each mutant was also transformed with the empty plasmid, pJN105. Bacterial strains were grown in the presence of 1 mg/mL lysozyme for 16 h. To induce the gene expression, 0.01% L-arabinose was also added in each media. Aliquots of each culture were diluted and plated for CFU quantification. Bacterial cultures were performed in triplicate, and the mean ± SD values are displayed. ^*^P < 0.05 vs. CFU of each mutant harboring pJN105.

When wild type copy of *bamB* or *fabY* gene was expressed by the arabinose-inducible promoter, each mutant became fully resistant to lysozyme treatment (Figure 3). These results further confirm that the lysozyme sensitivities observed in the mutants are indeed caused by the deletion of *bamB* or *fabY* gene.

### Lysozyme-sensitive mutants are also susceptible to treatment with airway mucus secretion

**Figure 4:**
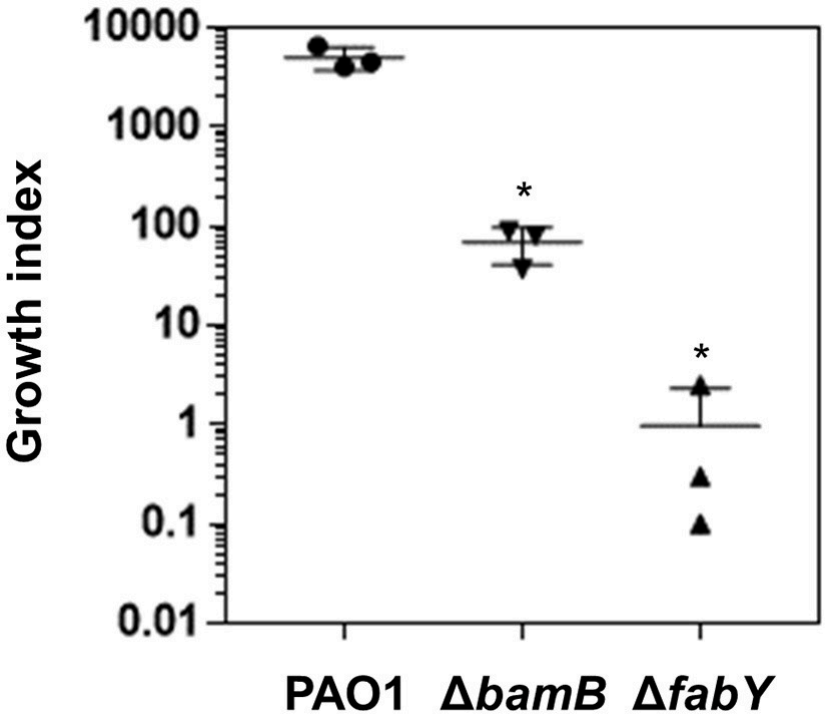
**Bacterial growth in airway mucus secretion (AMS) prepared from primary culture of human tracheal tissues**. The wild type PAO1 and its two mutants, Δ*bamB* and Δ*fabY*, were grown in three separate AMS preparations for 16 h. Aliquots of each culture were diluted and plated for CFU quantification. The values for CFU _in16h_ / CFU _inoculum_ were calculated and plotted as the growth index. Bacterial cultures were performed in triplicate, and the mean ± SD values are displayed. ^*^P < 0.05 vs. CFU of PAO1 cells.

Airway mucus secretion (AMS) plays a role in the host innate defense and contains various antimicrobial components including lysozyme (Gi et al., 2015). To examine bacterial response to AMS, we treated the bacterial strains with AMS collected from primary cultures of three different human tracheal tissues. In each treatment, 10^3^ bacterial cells were inoculated and incubated for 16 h. After the treatments, the number of PAO1 cells increased to ~5 × 10^6^ cells (growth index of ~5,000), demonstrating the capability of wild type *P. aeruginosa* to propagate in AMS (Figure 4). The growth of the Δ*bamB* mutant was not as robust as that of PAO1. The average growth index of the mutant in AMS was ~68 (Figure 4). Of note, the growth of the Δ*fabY* mutant was more severely affected, yielding the growth index of ~1.0. Together, these results suggest that two lysozyme-sensitive mutants are less capable of proliferating in AMS, a frontline substance that *P. aeruginosa* encounters during the early stage of airway infection.

### *In vivo* infectivity of the lysozyme-sensitive mutants was ameliorated

**Figure 5:**
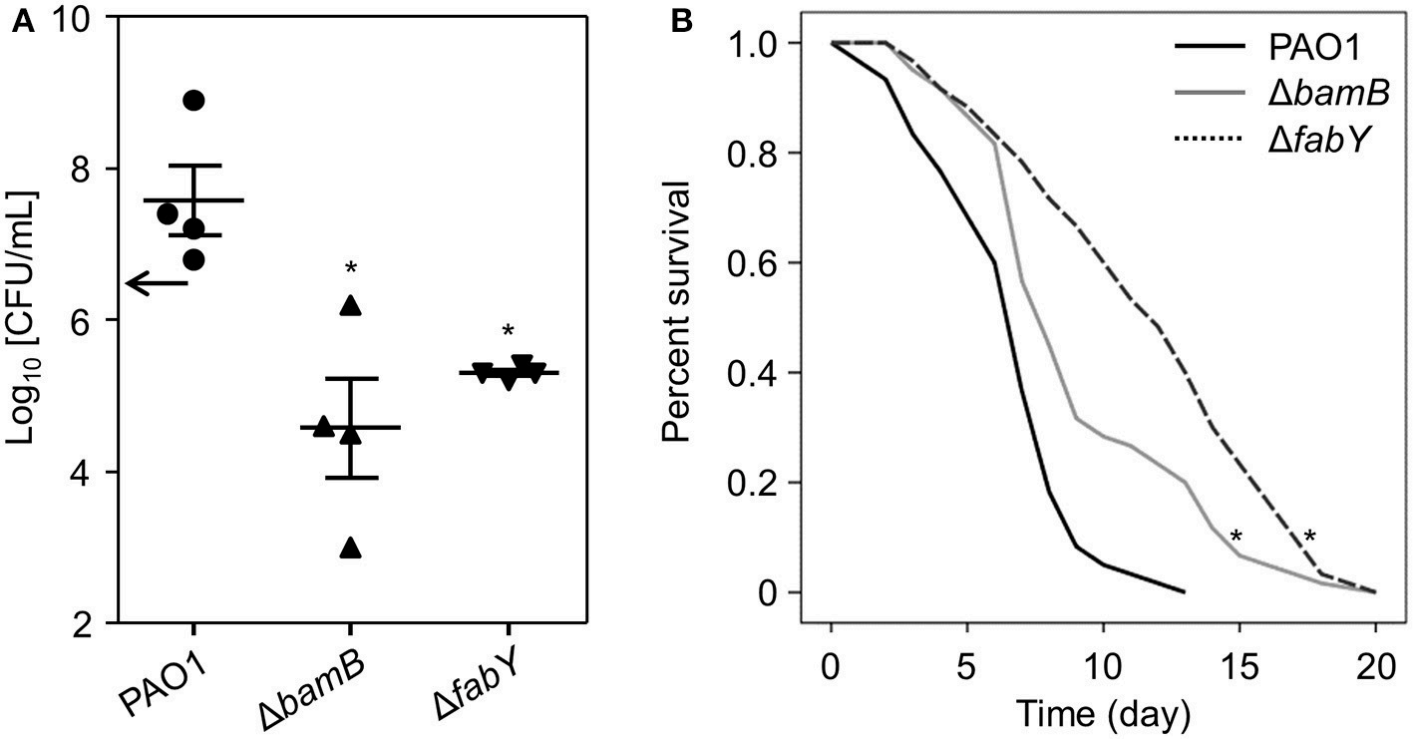
**Survival fitness and virulence of Δ*bamB* and Δ*fabY* mutants *in vivo*. (A)** Bacterial counts recovered from the left lung lobes. The infection dose was 5.0 × 10^6^ cells per mouse (black arrow on the y-axis). Mice were infected with the indicated bacterial strains for 16 h. ^*^P < 0.05 vs. CFU of PAO1 cells. **(B)** Survival curves of the *C. elegans* N2 strain fed with PAO1 (blue), Δ*bamB* (green), and Δ*fabY* (black). ^*^P < 0.05 vs. the survival rate of the PAO1-fed worms. In each group, 20 worms were used. Statistical significance was determined by log-rank analysis.

We next examined whether these mutants were also defective in establishing infections *in vivo*. To address this question, we utilized two different animal infection models: mouse and nematode. First, mice were intranasally infected with bacterial cells. At 16 h post-infection, mouse lung homogenates were prepared, and the bacterial cells were enumerated. Increased numbers of PAO1 cells were recovered following the 16 h infection (Figure 5A), demonstrating that wild type *P. aeruginosa* can replicate inside the mouse airway. By comparison, decreased numbers of Δ*bamB* and Δ*fabY* mutant cells were recovered, indicating a compromise in their ability to multiply inside a host airway (Figure 5A).

*C. elegans* is a nematode that has been widely used to study host-microbe interactions (Go et al., 2014). Furthermore, *C. elegans* is known to express 15 homologs of lysozyme that act as digestive enzymes for bacterial prey and, therefore, as key players for innate immunity (Mallo et al., 2002; Boehnisch et al., 2011). Given these characteristics, we postulated that *C. elegans* could serve as an appropriate model to study *in vivo* infectivity of lysozyme-sensitive mutants. As shown in Figure 5B, *C. elegans* lived significantly longer when fed Δ*bamB* and, to a greater extent, Δ*fabY* mutants; the average lifespans were 9.2 ± 0.5 (days) and 11.6 ± 0.6 (days), respectively. As expected, a considerably shorter lifespan (6.6 ± 0.3 days) was observed in worms grown by feeding on PAO1 cells (Figure 5B). Together, these results demonstrate that lysozyme-sensitive mutants are also less capable of establishing *in vivo* virulence.

### Bacterial cell membrane integrity was mildly and severely affected in Δ*bamB* and Δ*fabY* mutants, respectively

**Figure 6:**
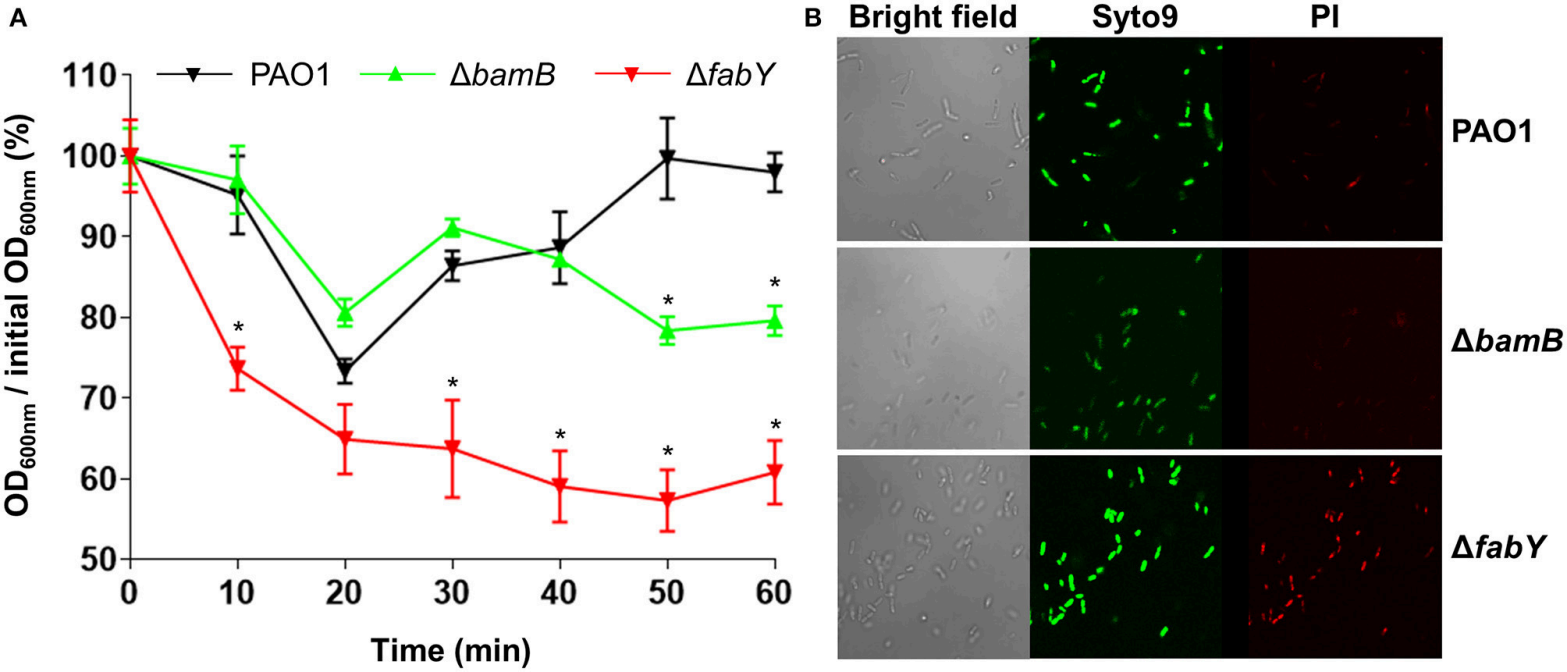
**Bacterial membrane integrity tests of two lysozyme-sensitive mutants**. **(A)** Bacterial cells resuspended in distilled water were monitored for their changes in OD_600_ values at 10 min intervals for 1 hr. Experiments were performed in triplicate, and the mean ± SD values are displayed. ^*^P < 0.05 vs. OD_600_ of PAO1 at each time point. **(B)** Each bacterial culture was stained with Syto9 and propidium iodide and visualized by confocal laser scanning microscope (CLSM) as described in Section Materials and Methods.

Because lysozyme targets bacterial peptidoglycan, we postulated that the increased lysozyme sensitivity of the mutants may be associated with altered bacterial cell membrane integrity. To address this issue, we analyzed how the lysozyme-sensitive mutants responded to hypo-osmotic stress. We also assessed the penetration of propidium iodide, a fluorescent dye that normally impermeable of healthy bacterial membrane, into bacterial cells of each strain. When PBS-washed bacterial cells were resuspended in distilled water, a marked and persistent drop in OD_600_-values were observed in the Δ*fabY* mutant over 1 h time period (Figure 6A). In contrast, bacterial cell density monitored by measuring OD_600_-values was decreased only in the later stage of the experiment for the case of the Δ*bamB* mutant (Figure 6A). Consistent with the hypo-osmotic resistance result, Δ*fabY* mutant cells were more permeable to propidium iodide, a red fluorescent dye that only stains bacterial nucleic acids when the membrane integrity was compromised. Together, these results demonstrate that bacterial membrane integrity is affected, especially in the Δ*fabY* mutant and such a defect likely accounts for the enhanced sensitivity (i) to the AMS treatment (Figure 4) and (ii) inside the *C. elegans* (Figure 5B).

### Δ*bamB* mutant is extremely sensitive to antibiotics that target cell wall synthesis

**Figure 7:**
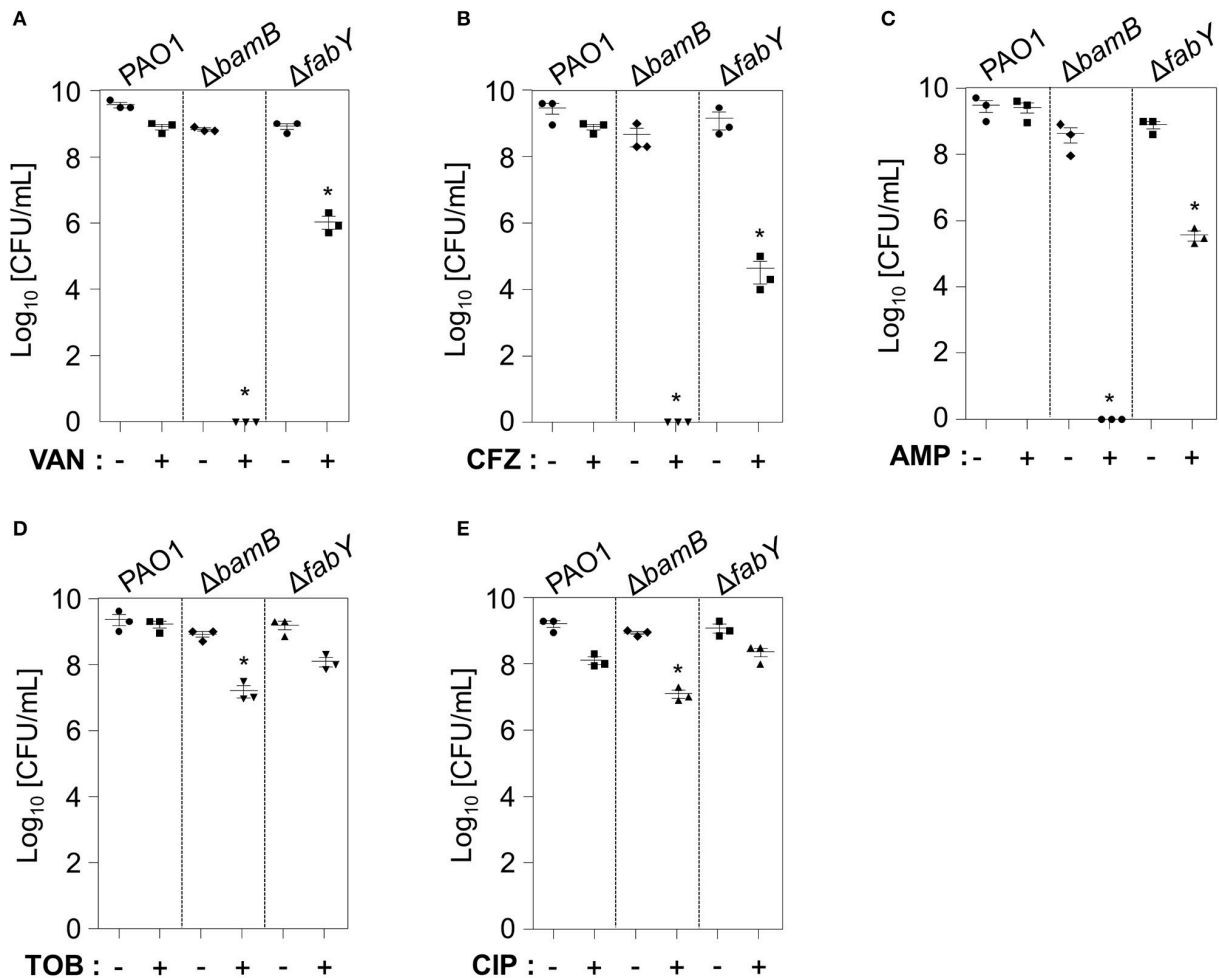
**Antibiotic susceptibility of two lysozyme-sensitive PAO1 mutants**. PAO1, Δ*bamB*, and Δ*fabY* mutants were treated in LB for 16 h with 100 μg/mL Vancomycin **(A)**, 0.5 μg/mL Ceftazidime **(B)**, 25 μg/mL ampicillin **(C)**, 0.2 μg/mL tobramycin **(D)**, or 0.1 μg/mL ciprofloxacin **(E)**. After a16-h incubation, aliquots from each culture were diluted and plated for CFU enumeration. ^*^P < 0.05 vs. CFU of untreated cultures. Antibiotic concentrations that do not affect PAO1 growth were chosen for analysis.

We then explored whether lysozyme-sensitive mutants also exhibited elevated sensitivity to antibiotic treatments. To examine bacterial responsiveness to antibiotics of diverse classes, we tested vancomycin (Day et al., 1993), ceftazidime (O'Callaghan, 1986), ampicillin (Ghobashy and Chiori, 1984), tobramycin (Hoff et al., 1974), and ciprofloxacin (Roy et al., 1983), the first three of which target cell wall synthesis. Tobramycin and ciprofloxacin inhibit protein synthesis and DNA replication, respectively. At the concentrations tested, PAO1 was either unaffected or only mildly affected (Figures 7A–E). Importantly, the Δ*bamB* mutant completely lost its viability in response to treatment with vancomycin, ceftazidime, and ampicillin (Figures 7A–C). The viability of the Δ*fabY* mutant was also decreased about 100~1,000 fold in response to the same treatment (Figures 7A–C). The degree of growth inhibition in these two mutants was not as great when treated with tobramycin and ciprofloxacin (Figures 7D,E). Collectively, these results suggest that (i) the Δ*bamB* mutant is exceptionally sensitive to cell wall-targeting antibiotics and, therefore, (ii) BamB may be an effective target in the elimination of *P. aeruginosa*.

## Discussion

Recalcitrant *P. aeruginosa* infection remains a huge threat to public healthcare. Increasing numbers of metallo-β-lactamase-producing clinical isolates have been identified around the world (Corvec et al., 2006; Chin et al., 2011; Jovcic et al., 2011; Piyakul et al., 2012; Van der Bij et al., 2012), and strains exhibiting resistance to colistin, a “last hope” antibiotic, have also been isolated (Lee et al., 2014). Therefore, novel anti-Pseudomonas strategies that do not generate escape mutants are required. We postulate that one such approach is to exploit lysozyme, an anti-bacterial innate immunoprotein that is abundantly present in the human airway. In this study, we identified PAO1 mutants that became sensitive to lysozyme treatment and provided the clinical perspectives to control *P. aeruginosa* infections.

The outer membrane of Gram-negative bacterial cells is a physical barrier that prevents lysozyme from gaining access to the peptidoglycan layer. EDTA-treated *P. aeruginosa* was found to be sensitive to lysozyme; when grown with Mg^2+^, the effect of EDTA-mediated increased susceptibility to lysozyme was reversed (Witholt et al., 1976; Ayres et al., 1998). These results suggest that outer membrane integrity is important for lysozyme resistance. In this respect, it is not surprising that lysozyme-sensitive mutants were also susceptible to antibiotics that target peptidoglycan synthesis. Of particular interest, however, is the extreme sensitivity of the Δ*bamB* mutant against cell wall-targeting antibiotics (Figure 7). Complete loss of viability was observed in response to treatment with vancomycin, ceftazidime, or ampicillin. These results demonstrate the potential of BamB as a drug target, the inhibition of which could result in efficient elimination of *P. aeruginosa* via a low dose of cell wall-targeting antibiotics. The crystal structure of the BamB indicates that it contains (i) a β-propeller fold with a central pore region and (ii) protruding loops that mediate its association with BamA (Jansen et al., 2012). BamA homologs in diverse bacterial species are considered essential for viability (Voulhoux et al., 2003; Gentle et al., 2004; Jansen et al., 2012). Given that BamB is an outer membrane protein, we can seek to identify a potential inhibitor that binds to the central pore region of BamB, thereby interfering with its function. Alternatively, the association of BamB with BamA could be targeted for inhibition. Mori and colleagues showed that peptides homologous to a portion of BamA-binding region of BamD can increase the efficacy of antibiotic-mediated killing of *P. aeruginosa* (Mori et al., 2012). It will be important to ask whether these peptides can also potentiate bactericidal action of lysozyme *in vitro* and inside the patient's airway as well.

The Δ*fabY* mutant produced hypoacylated lipid A and this change was proposed to be responsible for the increased antibiotic sensitivity (Six et al., 2014). Our result in Figure 6 shows that the Δ*fabY* mutant is more vulnerable to hypo-osmotic stress than Δ*bamB*. On the other hand, Δ*bamB* mutant cells are far more sensitive than Δ*fabY* to cell wall-targeting antibiotics (Figure 7). These findings suggest that alterations in fatty acid synthesis or outer membrane protein assembly may lead to unique cell surface changes, which result in distinct consequences. Importantly, both Δ*bamB* and Δ*fabY* mutant are equally susceptible to the treatment with 1.0 mg/ml lysozyme (Figure 2). We therefore hypothesize that lysozyme can be widely used in combination with any intervention that targets bacterial cell surface either at the lipid or protein level. We anticipate that the results provided here will stimulate future investigations.

Cerulenin, an anti-fungal agent, inhibits fatty acid biosynthesis by inhibiting β-ketoacyl-acyl carrier protein synthetase in *E. coli* (Buttke and Ingram, 1978). PAO1 FabY protein, however, exhibits low homology to the *E. coli* counterpart. Our experiments demonstrated that cerulenin at up to 200 μg/ml does not inhibit *P. aeruginosa* growth (data not shown). To date, a wide range of fatty acid synthesis inhibitors have been identified. These include isoniazid (Chan and Vogel, 2010), triclosan (Wright and Reynolds, 2007), Irgasan (Nishi et al., 2016), Kaempferol (Thors et al., 2008), Quercetin (Zhao et al., 2014a), Apigenin (Brusselmans et al., 2005), 1,2,3,4,6-Penta-O-galloyl-β-D-glucose (Zhao et al., 2014b), and Epigallocatechin gallage (Brusselmans et al., 2005). Although many of these inhibitors are proved to be effective in eukaryotic cells, these have not been actively tested in bacterial cells. It is worthwhile to examine whether any of these inhibitors can exert synergistic effects on inhibiting *P. aeruginosa* growth, when used together with lysozyme.

In conclusion, this study uncovered a common aspect of two different *P. aeruginosa* mutants with pleiotropic phenotypes. Our results show that interventions that affect fatty acid synthesis or outer membrane protein assembly may facilitate the eradication of *P. aeruginosa* with the aid of physiological concentration of lysozyme. Insights gleaned from this study will be useful in devising new strategies to combat one of the most resistant infections.

## Author contributions

KML and SY conceived, designed, and coordinated the study. KML, KL, JG, IP, JS, JC, and HK performed the experiment and interpreted the data. KML, KL, and SY wrote the manuscript. All the authors participated in discussions of the results and reviewed the final draft.

## Funding

This work was supported by grants from the National Research Foundation (NRF) of Korea, funded by the Korean government (2014R1A2A2A01002861, 2014R1A4A1008625, and 2014R1A1A2059520). This work was also made possible by a grant from the Korea Healthcare Technology R&D Project of the Ministry for Health, Welfare, and Family Affairs (HI15C0694).

### Conflict of interest statement

The authors declare that the research was conducted in the absence of any commercial or financial relationships that could be construed as a potential conflict of interest.
