# Supplementary figures and images for: A Genetic Screen Reveals Novel Targets to Render Pseudomonas aeruginosa Sensitive to Lysozyme and Cell Wall-Targeting Antibiotics
Source: Front Cell Infect Microbiol. 2017 Mar 1;7:59. doi: 10.3389/fcimb.2017.00059 (PMC5331053; doi:10.3389/fcimb.2017.00059)

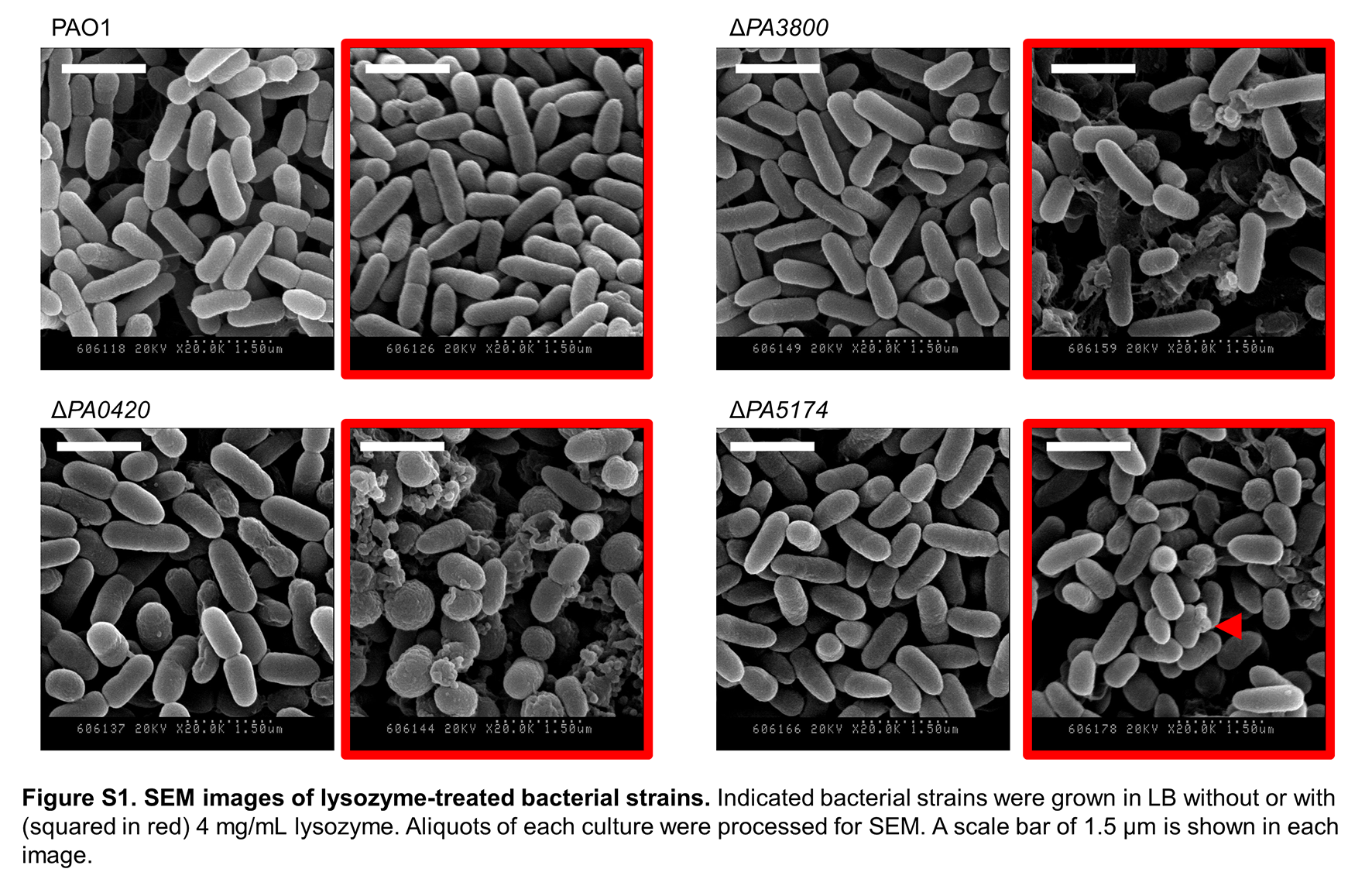

Supplement: Supplementary file 1 [file Image1.TIF]

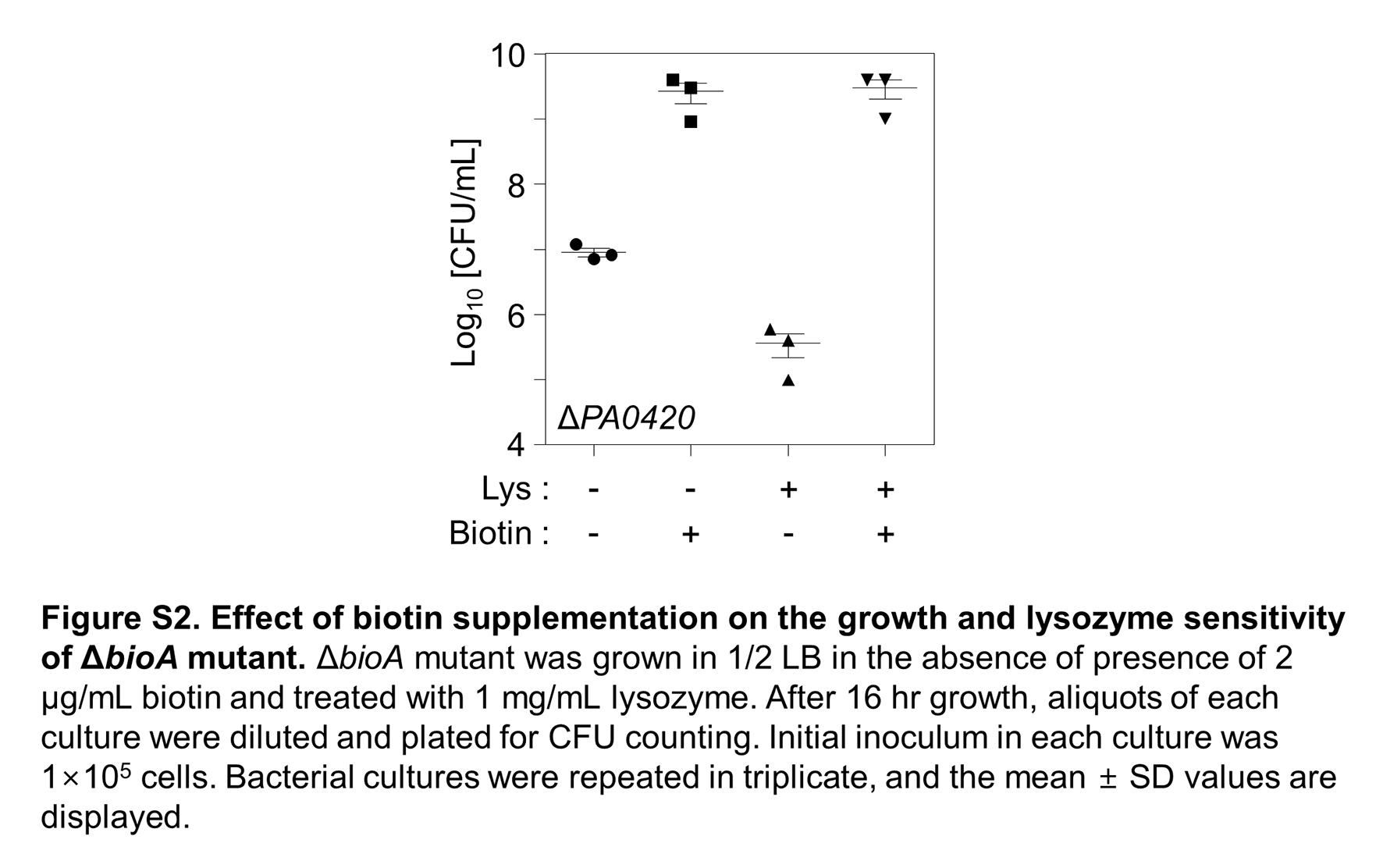

Supplement: Supplementary file 2 [file Image2.TIF]
